# *Inonotus rickii* (Agaricomycetes, Hymenochaetaceae) in Brazilian Cerrado: Expanding Its Geographic Distribution and Host List

**DOI:** 10.3389/fmicb.2021.647920

**Published:** 2021-03-09

**Authors:** Lucas Leonardo-Silva, Ahmed M. Abdel-Azeem, Solange Xavier-Santos

**Affiliations:** ^1^Basic, Applied and Scientific Dissemination Micology Laboratory (FungiLab), Goiás State University, Anápolis, Brazil; ^2^Department of Botany and Microbiology, Faculty of Science, Suez Canal University, Ismailia, Egypt

**Keywords:** Brazilian Savanna, Canker, decay, phytopathogen, poroid fungi

## Abstract

*Inonotus rickii* (Pat.) Reid (Agaricomycetes: Hymenochaetaceae) is a poroid fungus characterized by the expressive production of chlamydospores, *in vivo* and *in vitro*, especially during its anamorphic stage. The species plays important ecological roles, standing out as a phytopathogen, affecting several species of ornamental and wild trees, mainly in tropical and subtropical regions. The infected trees develop canker and white rot of the wood, showing symptoms of reduced vegetative vigor and decline of leaves and branches which causes death in some cases. The first record of *I. rickii* for the Cerrado biome (Brazilian Savanna) and the first record as causal agent of canker in *Schinus molle* L. in Brazil is reported here. In addition, we present a checklist of its worldwide geographical distribution and known hosts, from an extensive bibliographic search in Google Scholar, SciELO, Scopus, and Web of Science databases. The species is widespread in tropical and subtropical zones; common in the American continent, especially in Central and South America and the Mediterranean region, and rare in temperate zones. We found specimens growing in both living and dead hosts, totalizing 70 species of hosts, distributed in 43 genera and 22 families. Of these, *Acer negundo* L. (10.5%), *Celtis australis* L. (6.5%), and *Platanus acerifolia* (Aiton) Willd. (4.8%), and the Fabaceae (30%), Fagaceae (10%), and Sapindaceae (8.6%) families were the most frequent. We present morphological descriptions and illustrations, as well as the growth characteristics in culture medium. Our study expands the known geographical distribution of *I. rickii*, including the Cerrado biome, as well as its structural, physiological characteristics, and its hosts.

## Introduction

*Inonotus rickii* (Pat.) Reid is a poroid fungus that belongs to the family Hymenochaetaceae, class Agaricomycetes. It was described in South America in 1896 as *Ptychogaster cubensis* Pat., from a review of specimens from Cuba by Patouillard (1896). However, in 1908, the author observed that the species should be the anamorphic stage of a poroid fungus collected by Ricki in Brazil, recognized as *Xanthochrous rickii* Pat. (Patouillard, 1908); later the nomenclature was changed to *Polyporus rickii* (Pat.) Sacc. and Trotter (Saccardo and Trotter, 1912). In 1957, the species was recognized by Reid as *I. rickii* (Reid, 1957), currently accepted nomenclature, and its anamorphic stage as *P. cubensis*.

The relationship between the two life stages of the species has been confirmed by experimental studies carried by Davidson et al. (1942) and Stalpers (2000) and by molecular analysis of specimens collected from different geographical origins (Gottlieb et al., 2002; De Simone et al., 2011). *I. rickii* has a wide distribution in tropical, subtropical, and Mediterranean zones (Mazza et al., 2008; Ramos et al., 2008), where it is frequently found in trees in urban environments, mainly in its anamorphic stage (Mazza et al., 2008), which is considered a potential phytopathogen.

As a pathogen of woody plants, *I. rickii* infects branches and stems, causing canker and wood decay (Mazza et al., 2008; Annesi et al., 2010; De Simone et al., 2011). Adhesion and colonization of the substrate occurs due to the degradation of wood components, especially cellulose, hemicellulose and lignin, due to taxon enzymatic activity (Robles et al., 2014). When parasitizing the host, *I. rickii* can reach the heartwood, sapwood, and cambium, as well as provoke deep lesions resulting from the death of the bark tissues, characterizing the canker (Ramos et al., 2008; Annesi et al., 2010). Infected trees may show reduced vegetative growth, crown and branches decline and sparse foliage, which leads, in some cases, to death (Mazza et al., 2008; Ramos et al., 2008; Annesi et al., 2010).

The knowledge of this species-host began with reports describing the presence of a powdery mass of abundant ferruginous chlamydospores, which characterizes the anamorphic stage of the taxon in different plant hosts, but without mentioning its pathogenicity (Davidson et al., 1942). However, in the beginning of 2000, the diseases symptoms and pathological aspects became more evident in Europe. In Italy, the species was recorded in public gardens and wooded boulevards on *Acer negundo* L. and *Albizia julibrissin* Durazz. (Mazza et al., 2008; Annesi et al., 2010), and in Portugal, on *Celtis australis* L. (Ramos et al., 2008) causing serious damage or death of tree. Other reports in Chile (Sepúlveda et al., 2016), China (Cui et al., 2014), and Egypt (Shehata and Abdel-Wahab, 2013) also presented a similar situation regarding *Schinus molle* L., *Acacia richii*, A. Gray, *Citrus* spp. and *Vitis* spp. respectively. Based on the morphology of the basidiome and species of the host plants, the fungus called by different popular names e.g., “mapúa cheap,” which means “mascarilla de la mapúa,” in the Wayuu indigenous community of Colombia (Villalobos et al., 2017), or “Florcita de espinillo,” “Florcita de molle,” “Florcita de palo,” “Hongo de espinillo,” “Hongo de molle,” and “Hongo de palo,” in La Paz, Córboda, Argentina (Flamini et al., 2015).

In Brazil, information on *I. rickii* is mystery or fragmented and restricted to the holotype with unknown localities (Patouillard, 1908) and to occurrence records in the Caatinga biomes, on *Spondias* sp. (Umbuzeiro) (Drechsler-Santos et al., 2013; Maia et al., 2015); Atlantic Forest and Pampa without host identification (Campos-Santana and Loguercio-Leite, 2010; Maia et al., 2015). Here we report for the first time *I. rickii* in the Cerrado biome, which constitutes the first record of this species causing canker disease in *S. molle*. In addition, we present detailed morphological descriptions of both anamorphic and teleomorphic stages supported with illustrations, and updated checklist of its worldwide geographical distribution and known hosts up till now.

## Materials and Methods

### Study Area

**FIGURE 1:**
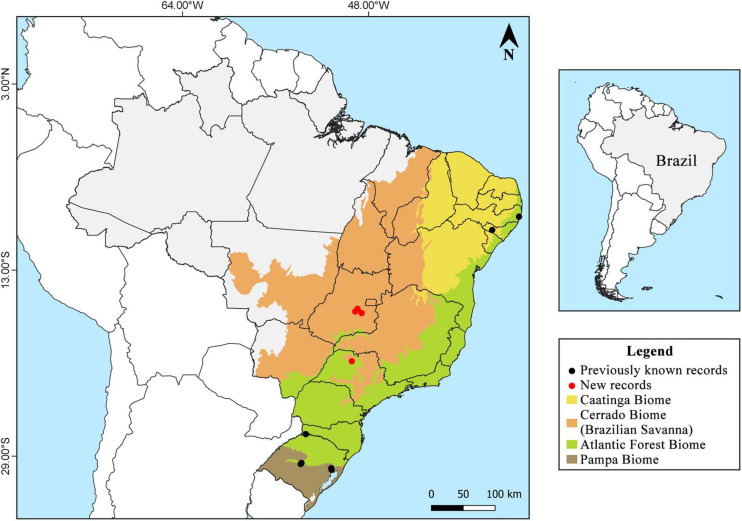
Study area and geographical distribution of *Inonotus rickii* in Brazil.

Samples were collected during the period from 2001 to 2020 in three conservation units of the Cerrado biome: the Estação Ecológica do Noroeste Paulista (EENP) (20°50′55″ S, 49°26′53″ W), located between the municipalities of São José do Rio Preto and Mirassol, São Paulo; the Floresta Nacional (FLONA) de Silvânia (16°38′30″ S, 48°39′02″ W), located in the municipality of Silvânia, Goiás; the Parque Estadual Altamiro de Moura Pacheco (PEAMP) (16°33′12″ S, 49°8′50″ W), inserted in the municipalities of Goianápolis, Goiânia and Nerópolis, Goiás, and urban areas (16°19′38″ S, 48°57′11″ W) in the municipality of Anápolis, Goiás, which are surrounded by several Cerrado fragments (Figure 1).

The Brazilian Cerrado is considered the richest savanna in the world and is the dominant biome in the Central Plateau of Brazil which covers part of the North, Northeast, South, and Southeast regions, representing approximately 25% of the country’s territory. The Cerrado characterized by a rainy tropical climate, with high temperatures in summer and dry winter, this biome is known for its diverse landscapes represented by a vegetation mosaic, which includes forest, savanna, and grassland formations, where trees, shrubs, and undergrowth predominate, respectively (Klink and Machado, 2005; Ribeiro and Walter, 2008; WWF, 2015). Despite its great diversity, only 61% of the original Cerrado vegetation remains preserved (Sano et al., 2010). Sampling of our target species occurred in mesophilic forest (semideciduous dry forest) in which there is a predominance of tree species and canopy formation, occurring in interfluves and rich soil presented different levels of deciduous vegetation in the dry season (Ribeiro and Walter, 2008).

## Morphological Characterization

Collected samples were dried at 40°C in oven and deposited in the Herbarium of the Universidade Estadual de Goiás (HUEG) and the Universidade Estadual Paulista (SJRP). Micro and macrophenotypic identification of basidiomata were carried out according to the relevant identification keys. For macroscopic characterization, the shape, consistency, color, dimension, and number of pores per mm of basidiomata were considered. For the description of the microscopic criteria, cross sections through the basidiomata in distilled water and 3% KOH were microscopically examined. Hyphal structure, hymenial setae, setal hyphae, basidia, basidiospores, and chlamydospores and their ornaments were examined according to Ryvarden (2004, 2005) and Ramos et al. (2008). Melzer reagent was used to test the amyloid reaction of the microstructures (Kirk et al., 2008). All microscopic criteria were observed by Olympus CX31 optical microscope (1000×) magnification, and the measurements were performed using the Piximètre software version 5.10 R 1541 (Henriot and Cheype, 2017). Abbreviations used for measurements of basidiospores and chlamydospores are: *Q* = quotient between length and width, Qm = medium value of *Q* and, *N* = number of measured structures. All photographs and measurements were made using 3% KOH as mounting medium. The color indication for all evaluated characters was based on Kornerup and Wanscher color cards (Kornerup and Wansher, 1978).

Fresh collected samples were plated out on potato dextrose agar (PDA) supplemented with 0.025 g/ml^–1^ of chloramphenicol as bactericidal and incubated in a BOD incubator at 25°C. Recovered colonies were characterized based on macro (shape, color, texture, and presence of exudates) and micromorphological criteria (hyphae, setal hyphae, and chlamydospores) according to relevant identification keys. The cultures were preserved according to Castellani (1963) and deposited in the collection of fungal cultures of the Laboratório de Micologia Básica, Aplicada e Divulgação Científica (FungiLab) of the Universidade Estadual de Goiás, Brazil.

### Checklist of Geographical Distribution and Hosts

We collected the data from an extensive bibliographic search of Google Scholar^1^, SciELO^2^, Scopus^3^, and Web of Science^4^ by using different keywords: “*Inonotus rickii*” OR “*Polyporus rickii*” OR “*Ptychogaster cubensis*” OR “*Xanthochrous rickii*.” Our data included all published articles, books, chapters, and abstracts available for access. For the worldwide geographical distribution of the species, we consider the specific location described by the authors and the geographic coordinates; when not available, they were obtained using the Latlong coordinate system (Latitude and Longitude Finder, 2020). The geographic distribution map was constructed using the Quantum GIS software (QGIS Development Team, 2020) and the climatic classification was determined between the North and South zones of both hemispheres as: tropical between 23°27′, subtropical 23°27′ and 46°54′, temperate 46°54′ and 66°33′, and polar 66°33′ and 90°, respectively (Peel et al., 2007; Eccles et al., 2019).

For the host checklist, we considered the absolute frequency the number of times the species was reported and relative frequency the quotient between the absolute frequency and the total of citations. The system of nomenclature, hierarchical classification and the name authority of the plant species followed The plant list (The Plant List, 2020).

## Results

### Taxonomic Treatment

**FIGURE 2:**
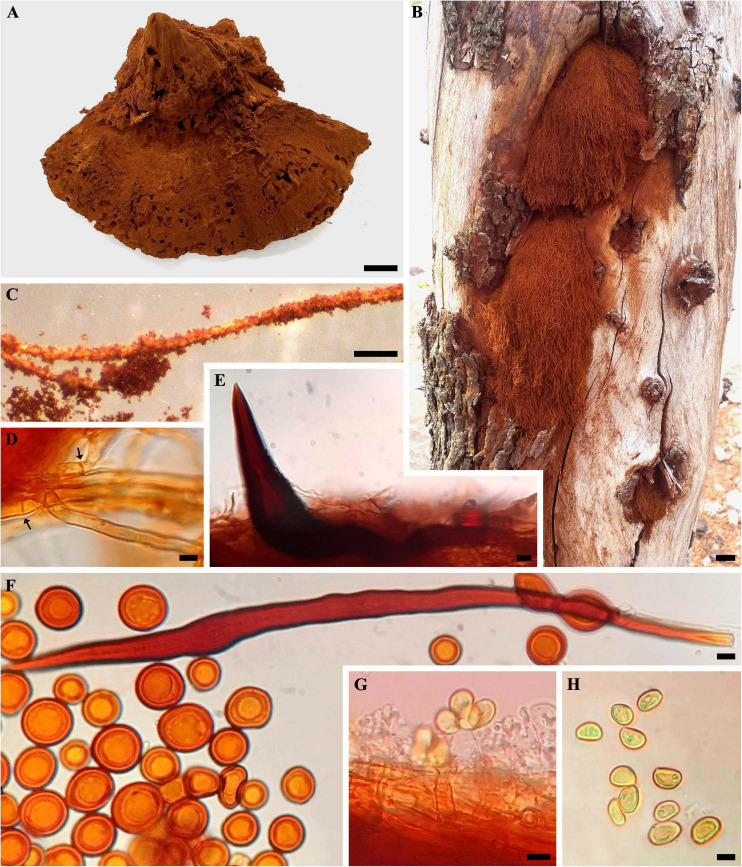
*Inonotus rickii*. **(A)** Basidiome (HUEG 12067). **(B)** Anamorphic stage (HUEG 12062). **(C)** Chlamydospores attached to setal hyphae. **(D)** Generative hyphae, hyaline with simple septate (arrow). **(E)** Hymenial setae. **(F)** Setal hyphae and abundant chlamydospores. **(G)** Basidia hyaline, with projection of four sterigmatic structures and attached basidiospores. **(H)** Basidiospores. Bar = 1 cm **(A,B)**; 5 mm **(C)**; 5 μm **(D–H)**.

**FIGURE 3:**
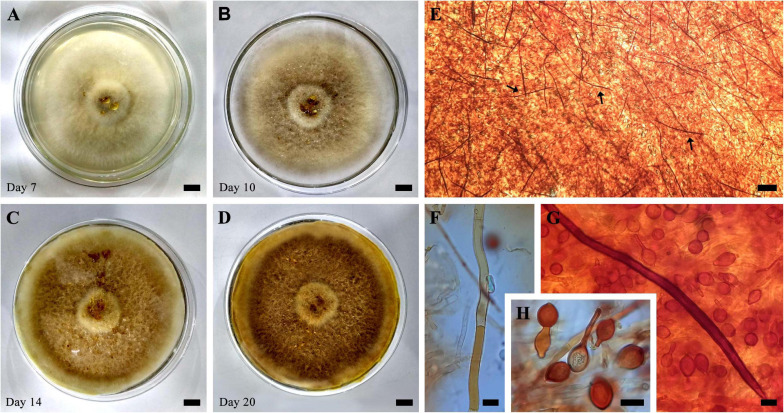
**(A–D)** Evolution of growth in cultivation (SXS 37) in PDA at 25°C for 20 days. **(E)** Mycelial trama showing abundant setal hyphae (arrows). **(F)** Generative hyphae. **(G)** Setal hyphae and chlamydospores. **(H)** Chlamydospores. Bar = 1 cm **(A–D)**; 100 μm **(E)**; 10 μm **(F–H)**.

*Inonotus rickii* (Pat.) D. A. Reid, Kew Bull. [12](2): 141 (1957). (Figures 2, 3).

≡ *Xanthochrous rickii* Pat., Bull. Soc. Mycol. France 24(1): 6 (1908).

### Description

Teleomorphic stage–basidiomata annual, 7.0–11 × 6.0–7.8 cm in size, solitary or aggregated in the form of semicircular shelves, sessile, pileate, and strongly attached to the substrate. Pileus applanate to ungulate, margin obtuse to undulate, soft and spongy consistency when fresh and firm and crumbly when dry. Pileal surface covered by a dense powdery layer formed by reddish brown chlamydospore (8D7). Pore surface also covered by chlamydospore, circular to angular pores, 2–4 pores per mm, thin and lacerated. Thick context and positive KOH reaction. Anamorphic stage–semicircular or cushion-shaped, soft and robust, velvety to the touch, reddish orange (8B8) when young and reddish brown (8E7) when mature, structured by a dense mass of chlamydospore attached to hyphae, forming a structure similar to capillitium, which facilitates dispersion by anemophilia.

Hyphal system monomitic, with generative hyphae, hyaline, septate, ranging from brownish yellow (5C7) to brownish orange (6C8), thin-walled to thick-walled, occasionally branched, 3.0–6.6 μm in diam. Setal hyphae abundant in context, lanceolate, yellowish brown (5D5), thick-walled with a large lumen, with pointed apex, occasionally parallel to the hymenium, 8–20 μm in diam. and 110–200 μm long. Hymenial setae abundant, ventricular to subulate, dark brown (6F8), 14.3–63 × 5.0–18.4 μm, with thick-walled. Basidia hyaline, clavate to cylindric, 10 × 6.5 μm, with projection of four sterigmatic structures. Basidiospores abundant, subglobose to ellipsoid, (5.4) 5.9–8.7 (9.5) × (3.6) 4.3–6.2 (6.5) μm [*Q* = (1) 1.2–1.6 (2.1), Qm = 1.4, *N* = 50], golden yellow (5B7) to yellowish brown (5D5), dark brown (6F3), inamyloid, smooth with thick-walled. Chlamydospores abundant in the context, inamyloid, irregular, globose to subglobose, (7.4) 9.1–13.7 (15.5) × (6.9) 8.0–11.6 (13.9) μm [*Q* = 1.0–1.3 (1.9), Qm = 1.1, *N* = 50], orange (6A8) to reddish orange (7A8), dark brown (6F3), smooth with thick-walled.

In culture presents velutinous to cottony mycelium, occupying the entire length of the petri dish (90 × 15 mm) in 2 weeks of cultivation, yellowish white (2A2) when young, becoming grayish yellow (4B4) to dark yellow (4C8) when mature, production of exudates in the form of reddish orange droplets (8A8). Abundant production of chlamydospores of various shapes, occasionally attached to setal hyphae, also abundant and parallel in the hyphalic trama. The color and size characteristics of the cultures’ microstructures are in accordance with those observed in the basidiomata.

### Worldwide Geographical Distribution

**FIGURE 4:**
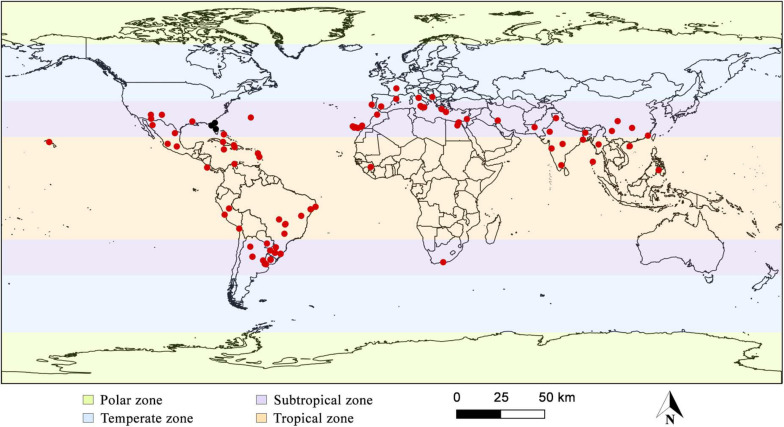
Global geographical distribution of *Inonotus rickii*. Black dots highlight samples that may belong to another species, according to De Simone et al. (2011) and Cui et al. (2014).

Widespread in tropical and subtropical zones, considered then pantropical; common on the American continent, especially in Central and South America and the Mediterranean region, rare in temperate zones. There are records of the species in Argentina, Bahamas, Bermuda Islands, Brazil, Chile, China, Colombia, Costa Rica, Cuba, Egypt, France, Greece, Guadeloupe, Guinea, Haiti, India, Iran, Israel, Italy, Jamaica, Martinique, Mexico, Montenegro, Morocco, Myanmar, Pakistan, Paraguay, Peru, Philippines, Portugal, South Africa, Spain, Taiwan, United States, and Uruguay (Figure 4; Seaver and Waterston, 1946; Jaquenoud, 1985; Kotlaba and Pouzar, 1994; Chang and Fu, 1998; Stalpers, 2000; Gottlieb et al., 2002; Melo et al., 2002; Ryvarden, 2005; Martínez, 2006; Mata et al., 2007; Ghobad-Nejhad and Kotiranta, 2008; Annesi et al., 2010; Campos-Santana and Loguercio-Leite, 2010; Ţura et al., 2010; De Simone et al., 2011; Ouabbou et al., 2012; Shehata and Abdel-Wahab, 2013; Valenzuela et al., 2013; Cui et al., 2014; García et al., 2014a; Robles et al., 2015a; Sepúlveda et al., 2016; Villalobos et al., 2017; Maubet, 2020; Tchoumi et al., 2020). Table 1 show detailed description of the species’ worldwide geographic distribution, which contains the exact sampling location of each specimen described in the literature data found.

**TABLE 1:** Referenced literature for the global geographical distribution of *Inonotus rickii*.

Country	Locality	References
Argentina	Acassuso, Buenos Aires Province	Gottlieb et al., 2002
	Argentina (location not specified)	Intini, 1988
	Buenos Aires City	Gottlieb et al., 2002; Mielnichuk and Lopez, 2007; Robles et al., 2011, 2012, 2015a,b
	Buenos Aires Province	Gottlieb et al., 2002
	Córdoba, Córdoba Province	Urcelay et al., 2012
	Gualeguay, Entre Ríos Province	Gottlieb et al., 2002
	La Plata, Buenos Aires Province	Gottlieb et al., 2002; Wright and Alberto, 2006; Murace et al., 2019
	Llavallol, Buenos Aires Province	Gottlieb et al., 2002
	Lomas de Zamora, Buenos Aires Province	Gottlieb et al., 2002
	Martínez, Buenos Aires Province	Gottlieb et al., 2002
	Rosario del Tala, Entre Ríos Province	Gottlieb et al., 2002
	Santo Tomé, Corrientes Province	Gottlieb et al., 2002
	Tucumán Province	Gottlieb et al., 2002
	Valle de Traslasierra, Córdoba Province	Flamini et al., 2015
	Yacanto, Córdoba Province	Gottlieb et al., 2002
Bahamas	Bahamas (location not specified)	Intini, 1988; Ryvarden, 2005
	Nassau	Davidson et al., 1942
Bermuda Islands	Bermuda Islands (location not specified)	Seaver and Waterston, 1946
Brazil	Anápolis, Goiás	Present study
	Bahia	Davidson et al., 1942; Stalpers, 2000
	Brazil (location not specified)	Davidson et al., 1942; Ryvarden, 1983; Intini, 1988
	Goiânia, Goiás	Present study
	Ipojuca, Pernambuco	Maia et al., 2015
	Mondaí, Santa Catarina	Campos-Santana and Loguercio-Leite, 2010
	Porto Alegre, Rio Grande do Sul	Campos-Santana and Loguercio-Leite, 2010
	Santa Maria, Rio Grande do Sul	Campos-Santana and Loguercio-Leite, 2010
	São José da Tapera, Alagoas	Drechsler-Santos et al., 2013
	São José do Rio Preto-Mirassol, São Paulo	Present study
Chile	Arica, Arica Province	Sepúlveda et al., 2016
China	Hainan Province	Cui et al., 2009; Dai et al., 2010; Yuan et al., 2015
	Jianyang, Sichuan Province	Zheng et al., 2011
	Panzhihua, Sichuan Province	Chen et al., 2014; Cui et al., 2014
Colombia	Uribia, Guajira	Villalobos et al., 2017
Costa Rica	Puntarenas, Cóbano	Mata et al., 2007
Cuba	Cuba (location not specified)	Murrill, 1919; Intini, 1988; Stalpers, 2000
Egypt	Beni Suef Province	Shehata and Abdel-Wahab, 2013
	Giza Province	Shehata and Abdel-Wahab, 2013
	Qalubyia Province	Shehata and Abdel-Wahab, 2013
France	France (location not specified)	Ryvarden, 2005
Greece	Crete, Iráklion	Kotlaba and Pouzar, 1994
	Monemvasia, Laconia	De Simone et al., 2011
Guadeloupe	Guadeloupe (location not specified)	Jaquenoud, 1985; Intini, 1988
Guinea	Guinea (location not specified)	Jaquenoud, 1985; Intini, 1988
Haiti	Haiti (location not specified)	Davidson et al., 1942; Intini, 1988
	Tortuga Island	Davidson et al., 1942
India	Andaman and Nicobar Islands, Manjari	Sharma and Mishra, 2015
	Calcutta, West Bengal	Davidson et al., 1942
	Dindigul, Tamil Nadu	Mowna Sundari et al., 2018
	India (location not specified)	Intini, 1988; Ryvarden, 2005
	Jodhpur, Rajasthan	Singh et al., 2013
	Pune, Maharashtra	Jagtap et al., 2018
	Western and Eastern Himalaya	Sharma and Mishra, 2015
Iran	Khuzestan Province	Ghobad-Nejhad and Kotiranta, 2008
Israel	Tel Aviv	Ţura et al., 2010
Italy	Catania, Sicily	Intini, 1988; Annesi et al., 2005, 2010; De Simone et al., 2011
	Palermo, Sicily	Jaquenoud, 1985; Venturella and Raimondo, 2004; Annesi et al., 2005, 2010; Venturella et al., 2006; De Simone et al., 2011
	Roma	Annesi et al., 2003, 2010, 2005; Mazza et al., 2008; De Simone et al., 2011
	Sicily	Ryvarden, 2005
Jamaica	Jamaica (location not specified)	Jaquenoud, 1985; Intini, 1988
Martinique	Fort-de-France	David and Rajchenberg, 1985; Jaquenoud, 1985; Intini, 1988
Mexico	Jalisco	Valenzuela et al., 2013
	Mexico City	Valenzuela et al., 2013
	Nuevo León	Valenzuela et al., 2013
	Sonora	Esqueda et al., 2010; Valenzuela et al., 2013
	Sonoran Desert, Nacapule Canyon	Raymundo et al., 2013
Montenegro	Budva	Kotlaba and Pouzar, 1994
Morocco	Forest of Mamora	Ouabbou et al., 2012
	Morocco (location not specified)	Intini, 1988
	Lima	Davidson et al., 1942
Myanmar	Myanmar (location not specified)	Jaquenoud, 1985; Intini, 1988
Pakistan	Pakistan (location not specified)	Jaquenoud, 1985; Intini, 1988
Paraguay	San Lorenzo	Maubet, 2020
Peru	Peru (location not specified)	Intini, 1988; Ryvarden, 2005
Philippines	Mindanao	Gottlieb et al., 2002
Portugal	Lisboa	Melo et al., 2002; Ramos et al., 2008
South Africa	Knysna, Western Cape Province	Tchoumi et al., 2017, 2020
Spain	Barcelona	De Simone et al., 2011
	Canary Islands, Rosa del Taro	García et al., 2014a
	Córdoba	De Simone et al., 2011
	Gran Canaria, Canary Islands	Vergara et al., 2016
	La Gomera, Canary Islands	García et al., 2014b
	La Palma, Canary Islands	García et al., 2014b
	Lanzarote, Canary Islands	García et al., 2014b
	Seville	Intini, 2002a, b; Intini and Tello, 2003
	Spain (location not specified)	Intini, 2002b
	Tenerife, Canary Islands	García et al., 2014b
Taiwan	Kinmen County	Chang and Fu, 1998
United States	Arizona	Gilbertson et al., 1974; Ryvarden, 2004, 2005
	Florida	Davidson et al., 1942; Gilbertson and Ryvarden, 1986; Intini, 1988; Barnard, 1993; Ryvarden, 2004, 2005
	Hawaii (location not specified)	Intini, 1988
	Hawthorne, Florida*	De Simone et al., 2011
	Jacksonville, Florida*	De Simone et al., 2011
	Levy County, Florida*	De Simone et al., 2011
	Louisiana	Davidson et al., 1942; Gilbertson and Ryvarden, 1986; Intini, 1988; Ryvarden, 2004, 2005
	New Mexico	Sanogo and Lujan, 2018
	Oahu, Hawaii	Davidson et al., 1942
	Tucson, Arizona	Gilbertson and Ryvarden, 1986
Uruguay	Uruguay (location not specified)	Martínez, 2006

*Records of uncertain identification, according to De Simone et al. (2011) and Cui et al. (2014).

### Geographical Distribution in Brazil

There are records in the Atlantic Forest areas in Pernambuco, Rio Grande do Sul and Santa Catarina, Caatinga areas in Alagoas and Bahia (record not shown in Figure 1, as the specific location was not found), Pampa areas in Rio Grande do Sul, and Cerrado areas (present study) in Goiás and São Paulo (Figure 1; Campos-Santana and Loguercio-Leite, 2010; Maia et al., 2015).

### Habitat and Substrate

We collect our specimens in the mesophilic forest in both stages of life, on dead wood and an unknown living host, and in urban areas in the anamorphic stage on living and dead *S. molle* trees. In the data set listed in the present review, we found 70 plant species distributed in 43 genera and 22 families (Table 2). Of these hosts, *A. negundo* (representing 10.5% of the total species), *C. australis* 6.5% and *Platanus acerifolia* 4.8%. On the family level Fabaceae came first by accounted 30% out of all host families followed by Fagaceae (10%) and Sapindaceae (8.6%). In the checklist, the host species referred as sp. or spp., were accounted for as a single species. Table 2 presents a complete list of *I. rickii* hosts.

**TABLE 2:** Checklist of hosts of *Inonotus rickii* reported in the literature.

Family	Host species	AF	RF (%)
Adoxaceae	*Sambucus nigra* L. (Kotlaba and Pouzar, 1994; Annesi et al., 2003, 2005; De Simone et al., 2011)	4	3.2
Anacardiaceae	*Lithraea molleoides* (Vell.) Engl. (Flamini et al., 2015)	1	0.8
	*Pistacia atlantica* Desf. (Vergara et al., 2016)	1	0.8
	*Schinus areira* L. (Urcelay et al., 2012)	1	0.8
	*Schinus molle* L. (Intini, 1988, 2002b**; Annesi et al., 2003; Intini and Tello, 2003**; García et al., 2014a; Sepúlveda et al., 2016)	5	4.0
	*Spondias* sp. (Drechsler-Santos et al., 2013)	1	0.8
Altingiaceae	*Liquidambar styraciflua* L. (Nakasone, 1993)	1	0.8
Casuarinaceae	*Casuarina cunninghamiana* Miq. (Gottlieb et al., 2002)	1	0.8
	*Casuarina equisetifolia* L. (Valenzuela et al., 2013)	1	0.8
	*Casuarina* spp. (Gilbertson and Ryvarden, 1986; Wright and Alberto, 2006)	2	1.6
Cannabaceae	*Celtis australis* L. (Kotlaba and Pouzar, 1994; Gottlieb et al., 2002; Intini, 2002b**; Melo et al., 2002; Annesi et al., 2003, 2015; Intini and Tello, 2003**; Mazza et al., 2008; Ramos et al., 2008)	8	6.5
	*Celtis iguanaea* (Jacq.) Sarg. as *Celtis ehrenbergiana* (Klotzsch) Liebm. (Urcelay et al., 2012)	1	0.8
	*Celtis spinosa* Spreng. (Gottlieb et al., 2002)	1	0.8
	*Celtis tala* Gillies ex Planch. (Wright et al., 1988, unpublished; Gottlieb et al., 2002)	2	1.6
	*Celtis* sp. (Wright and Alberto, 2006)	1	0.8
Euphorbiaceae	*Hevea brasiliensis* (Willd. ex A. Juss.) Müll. Arg. (Cui et al., 2009; Dai et al., 2010)	2	1.6
Fabaceae	*Acacia caven* (Molina) Molina (Flamini et al., 2015)	1	0.8
	*Acacia koa* A. Gray (Davidson et al., 1942)	1	0.8
	*Acacia melanoxylon* R. Br. (Gottlieb et al., 2002)	1	0.8
	*Acacia praecox* Griseb. (Urcelay et al., 2012)	1	0.8
	*Acacia richii* A. Gray (Cui et al., 2014)	1	0.8
	*Acacia visco* Griseb. (Urcelay et al., 2012)	1	0.8
	*Albizia julibrissin* Durazz. (Annesi et al., 2007; Mazza et al., 2008)	2	1.6
	*Albizia lebbeck* (L.) Benth. (Ghobad-Nejhad and Kotiranta, 2008)	1	0.8
	*Albizia* sp. (Pieri and Rivoire, 1996)	1	0.8
	*Cercidium* sp. (Gilbertson and Ryvarden, 1986)	1	0.8
	*Delonix regia* (Hook.) Raf. (Tura et al., 2009; Ţura et al., 2010; Jagtap et al., 2018; Maubet, 2020)	4	3.2
	*Gleditsia sinensis* Lam. (Zheng et al., 2011)	1	0.8
	*Haematoxylon brasiletto* H. Karst (Villalobos et al., 2017)	1	0.8
	*Parkinsonia aculeata* L. (Gilbertson et al., 1974; Jaquenoud, 1985)	2	1.6
	*Parkinsonia praecox* (Ruiz and Pav.) Hawkins (Villalobos et al., 2017)	1	0.8
	*Parkinsonia* spp. (Jaquenoud, 1985, 1987; Gilbertson and Ryvarden, 1986; Ryvarden and Gilbertson, 1993; Annesi et al., 2003)	5	4.0
	*Prosopis cineraria* (L.) Druce (Singh et al., 2013)	1	0.8
	*Prosopis juliflora* (Sw.) DC. (Singh et al., 2013; Villalobos et al., 2017)	2	1.6
	*Robinia pseudoacacia* L. (Mazza et al., 2008; De Simone et al., 2011; Annesi et al., 2015)	3	2.4
	*Styphnolobium japonicum* (L.) Schott (Urcelay et al., 2012)	1	0.8
	*Tamarindus indica* L. (Davidson et al., 1942; David and Rajchenberg, 1985**; Jaquenoud, 1987**)	2	1.6
Fagaceae	*Quercus laevis* Walter as *Quercus catesbaei* Michx. (Davidson et al., 1942)	1	0.8
	*Quercus cerris* L. (Annesi et al., 2005; Fodor and Hâruta, 2016)	2	1.6
	*Quercus laurifolia* Michx. (Gilbertson and Ryvarden, 1986)	1	0.8
	*Quercus nigra* L. (Davidson et al., 1942)	1	0.8
	*Quercus phellos* L. (Davidson et al., 1942)	1	0.8
	*Quercus* sp. (Gilbertson and Ryvarden, 1986)	1	0.8
	*Quercus geminata* Small as *Quercus virginiana* var. *geminata* (Small) Sarg. (Davidson et al., 1942)	1	0.8
Icacinaceae	*Apodytes dimidiata* E. Mey. ex Arn. as *Apodytes dimidiata* subsp. *dimidiata* (Tchoumi et al., 2017)	1	0.8
Juglandaceae	*Carya illinoinensis* (Wangenh.) K. Koch (Sanogo and Lujan, 2018)	1	0.8
Malvaceae	*Chorisia* spp. (Urcelay et al., 2012)	1	0.8
	*Brachychiton* sp. (Murace et al., 2019)	1	0.8
Moraceae	*Ficus carica* L. (Michailides, 2003)	1	0.8
	*Morus* spp. (Urcelay et al., 2012)	1	0.8
Myricaceae	*Morella cerifera* (L.) Small as *Myrica cerifera* L. (Davidson et al., 1942; Seaver and Waterston, 1946; Gilbertson and Ryvarden, 1986; De Simone et al., 2011*)	4	3.2
	*Myrica* sp. (Gilbertson and Ryvarden, 1986)	1	0.8
Oleaceae	*Fraxinus* sp. (Valenzuela et al., 2013)	1	0.8
	*Olea capensis* subsp. *macrocarpa* (C. H. Wright) I. Verd. (Tchoumi et al., 2017**Tchoumi et al., 2020**)	1	0.8
Platanaceae	*Platanus acerifolia* (Aiton) Willd. (Mazza et al., 2008; Robles et al., 2011, 2012, 2015a,b; Annesi et al., 2015)	6	4.8
	*Platanus* × *hispanica* Mill. ex Münchh as *Platanus* × *hybrida* Brot. (Intini, 2002b; Intini and Tello, 2003)	1	0.8
	*Platanus* spp. (Gottlieb et al., 2002; Intini and Tello, 2003; Wright and Alberto, 2006)	3	2.4
Rhamnaceae	*Ziziphus spina-christi* (L.) Desf. (Ghobad-Nejhad and Kotiranta, 2008)	1	0.8
Rutaceae	*Citrus* spp. (Shehata and Abdel-Wahab, 2013)	1	0.8
Salicaceae	*Dovyalis caffra* (Hook.f. & Harv.) Sim as *Aberia caffra* Hook.f. & Harv. (Annesi et al., 2005)	1	0.8
Sapindaceae	*Acer negundo* L. (Pegler, 1967; Wright et al., 1988; unpublished; Gottlieb et al., 2002; Intini, 2002a**Intini, 2002b**; Annesi et al., 2003, 2010, 2005; Intini and Tello, 2003**; Venturella and Raimondo, 2004; Venturella et al., 2006; Wright and Alberto, 2006; Mazza et al., 2008; De Simone et al., 2011; Murace et al., 2019)	13	10.5
	*Acer saccharinum* L. (Gilbertson and Ryvarden, 1986)	1	0.8
	*Acer* sp. (Gottlieb et al., 2002)	1	0.8
	*Koelreuteria paniculata* Laxm. (Annesi et al., 2007; Mazza et al., 2008)	2	1.6
	*Melicoccus bijugatus* Jacq. (Pegler, 1967)	1	0.8
	*Sapindus saponaria* L. (Melo et al., 2002)	1	0.8
Ulmaceae	*Ulmus* sp. (Murace et al., 2019)	1	0.8
Verbenaceae	*Aloysia citriodora* Palau as *Lippia citriodora* (Palau) Kunth (Gottlieb et al., 2002)	1	0.8
	*Lippia* sp. (Wright and Alberto, 2006)	1	0.8
Vitaceae	*Vitis* spp. (Shehata and Abdel-Wahab, 2013)	1	0.8

Absolute frequency (AF) = number of times that species was cited (*n* = 125); relative frequency (RF) = percentage of citations of the species in relation to the total of host species found.

*Records of uncertain identification, according to De Simone et al. (2011) and Cui et al. (2014).

**Articles that present the same data.

### Material Examined

BRAZIL. São Paulo: São José do Rio Preto-Mirassol, Estação Ecológica do Noroeste Paulista, 13/XI/2001, *Xavier-Santos, S. (SJRP 28714)*, teleomorphic stage, found growing on fragment of dead wood, unknown host, isolated in culture with voucher number *SXS 37*; Goiás: Silvânia, Floresta Nacional de Silvânia, 26/VI/2009, Xavier-Santos, S. (*HUEG 13945*), anamorphic stage, growing on living tree, unknown host; Goianápolis-Goiânia-Nerópolis, Parque Estadual Altamiro de Moura Pacheco, 08/VIII/2014, *Xavier-Santos, S. (HUEG 12067)*, teleomorphic stage, found growing on living tree, unknown host; Anápolis, urban area, 20/III/2011, *Xavier-Santos, S. (HUEG 13944)*, anamorphic stage, growing on living tree, unknown host; Parque Ipiranga, 19/IX/2018, *Leonardo-Silva, L. (HUEG 12062)*, anamorphic stage, growing on a dead ornamental tree of *S. molle*, isolated in culture with voucher number *SXS 641*; ibid, 05/XII/2018, *Leonardo-Silva, L. (HUEG 11993)*, anamorphic stage, growing on living *S. molle*; ibid, 27/V/2019, *Leonardo-Silva, L. (HUEG 12063)*, anamorphic stage, growing on dead *S. molle*; ibid, 09/V/2020, *Leonardo-Silva, L. (HUEG 12994)*, anamorphic stage, growing on living *S. molle*; ibid, 09/V/2020, *Leonardo-Silva, L. (HUEG 12996)*, anamorphic stage, growing on dead *S. molle*; ibid, 09/V/2020, *Leonardo-Silva, L. (HUEG 12997)*, anamorphic stage, growing on living *S. mole*; ibid, 10/XI/2020, *Leonardo-Silva, L. (HUEG 13946)*, anamorphic stage, growing on dead *S. mole*.

### Examined Reference Material

BRAZIL. Rio Grande do Sul: Santa Maria, 11/I/1993, *Gilberto Coelho (24–9) (ICN 097679)*; Alagoas: São José da Tapera, 17/VI/2008, *Drechsler-Santos (6) (URM 80418)*, on living tree, unknown host; ibid, 17/VI/2008, *Drechsler-Santos (21) (URM 80460)*, growing on living tree of *Spondias* sp. (Umbuzeiro); ibid, 17/VI/2008, *Drechsler-Santos (18) (URM 80582)*, an unknown living tree.

### Comments

The morphological characteristics observed in collected samples coincide with those described by many authors (Gottlieb et al., 2002; Melo et al., 2002; Ryvarden, 2005). Measurements of setal hyphae recorded by Ryvarden (2005) and Campos-Santana and Loguercio-Leite (2010) were up to 250 μm or more long. Although these measurements are similar to our samples, we recorded smaller hyphal setae ranged between 110 and 200 μm in the examined materials and pure cultures. *I. rickii* is similar to *Inonotus patouillardii* (Rick) Imazeki in the field but differentiating itself by the abundant presence of chlamydospores. Our specimens were collected during both rainy and drought periods which reflected the resistance to the climatic variations of the Cerrado.

## Discussion

Our study expands and shed the light on the geographic distribution of the species with special reference to the Cerrado biome and updated list of plant hosts. *I. rickii* is easily described by the massive production of chlamydospores *in vitro* and *in vivo*, mainly in the anamorphic stage, and the presence of setal hyphae in the context (Pegler, 1964; Melo et al., 2002; Ramos et al., 2008; Tura et al., 2009; Maubet, 2020). In all of our specimens we observe these characteristics. Furthermore, the taxonomic criteria observed in pure cultures are compatible with those described by Ramos et al. (2008) and Tura et al. (2009).

*Inonotus rickii* is considered one of the most nocive basidiomycete in urban trees (Ramos et al., 2008), parasitizing a large number of hosts, thus being able to form an effective biological corridor in the dissemination of the pathogen in this environment (Annesi et al., 2010). The infection of these trees has a great impact in some regions of the world, as ornamental trees when parasitized and with symptoms of the disease, lose their ornamental value and represent a great danger to other members of the population (Ramos et al., 2008).

In Europe, especially in Italy (Annesi et al., 2010) and Portugal (Ramos et al., 2008), it has been reported that canker caused in plant tissues has reduced the number of ornamental tree species in urban environments. In addition, in the Wayuu indigenous community in Colombia, it has been reported that the fungus has had an impact on local native vegetation, also parasitizing species of daily use, such as *Parkinsonia praecox* and *Haematoxylon brasiletto*, used for resin and paint extraction, respectively (Villalobos et al., 2017).

The most frequent host species of *I. rickii* are widely known as ornamental trees. However, some of the reported hosts are used as a food source (Barolo et al., 2014; Viveros Garciìa et al., 2018) or for extracting compounds for different applications (Sharma et al., 2018; Milena et al., 2019). This warns of the need to know the impact of *I. rickii* on species of local, regional and worldwide economics.

Our specimens were detected growing at the base of the stem and on main branches of living and dead trees, usually in the anamorphic stage. Only specimens SJRP 28714 and HUEG 12067 were sampled in their teleomorphic stage. Although the species produces reproductive structures in both stages of the life cycle, the teleomorphic phase occurs occasionally, with the anamorphic stage usually frequent (Mazza et al., 2008; Ramos et al., 2008).

The propagation of the species occurs mainly by the production and release of chlamydospores, which are more abundant in the anamorphic phase. At this stage, a structure sensitive to touch appears, which releases a large number of chlamydospores. On this occasion, in urban environments, dispersal is facilitated by tactile gardening activities in the maintenance of squares and parks, by anemophilia, by the contact of insects and other animals, including the flux of human traffic (Annesi et al., 2010). We observed that all specimens collected in an urban area (HUEG 11993, 12062, and 12063) were in the anamorphic stage and the dispersion of chlamydospores may have been facilitated by the activities mentioned before. Infection and development of the fungus occur when its spores (basidiospores or chlamydospores) are deposited on susceptible hosts, preferably due to the presence of dead wounds, scars, or fragments; these spores then germinate and promote the growth of the fungus within wood tissues (Phillips and Burdekin, 1989).

In the present study, we report for the first time occurrence of *I. rickii* on *S. molle* in Brazil. The fungus was found either as saprobic or as a pathogen, causing canker of the host plant. Belonging to the Anacardiaceae family, *S. molle* is an arboreal and perennial species, popularly known as pepper tree, pink pepper, American pepper, false pepper, aroeira salsa (Brazil) or aroeira mansa (Brazil). It is native to South America, but it was introduced and naturalized in several regions of the world, mainly as an ornamental in urban environments. Its great popularity as a cultivated plant is associated with its high tolerance to water and temperature availability, high growth rate and medicinal properties (Kramer, 1957; Goldstein and Coleman, 2004; Habte et al., 2020).

**FIGURE 5:**
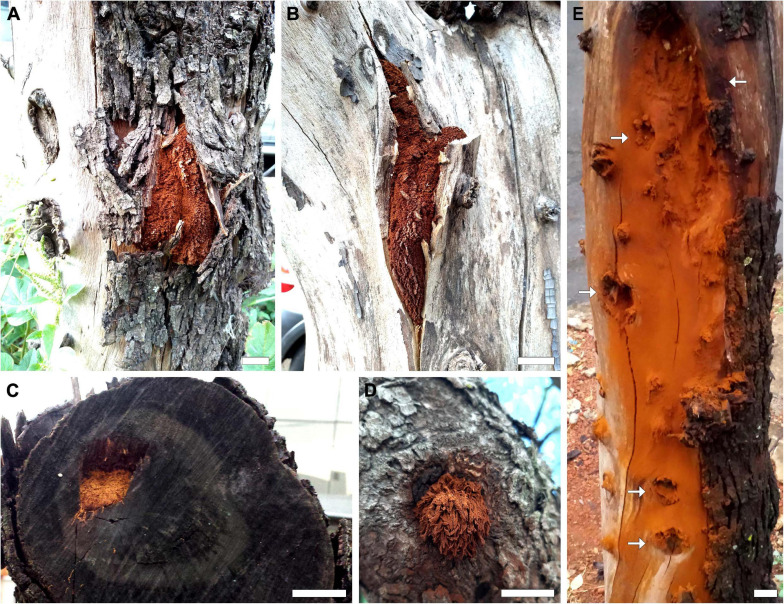
*Inonotus rickii* in its anamorphic stage on *Schinus molle*. **(A–C)** Fruiting projecting between the bark, bark and heartwood of dead tree. **(D)** Initial projection of the fruiting body on living tree. **(E)** Powdery, rust-colored coating on the wood, which was formed by the deposition of chlamydospores when removing fruiting; it is also possible to observe the lesions (arrows) caused by the infection along the trunk. Bar = 2 cm.

Anamorphic samples infected *S. molle* (HUEG 11993, 12062, and 12063) were developed over the trunk and main branches of the living and dead host. We observed that the individuals growing in the dead host (HUEG 12062 and 12063) occupied a large extension of the wood between the bark and bark, projecting over the crevices of the stem and reaching to the heart (Figure 5). On the other hand, samples that grew on a living host (HUEG 11993), initial canker development was observed, possibly due to the time of maturity of the fungus that was in the early stages of development (Figure 5). The presence of *I. rickii* in this plant has already been reported in Portugal (Melo et al., 2002), Chile, and Spain (Intini and Tello, 2003; Sepúlveda et al., 2016).

Parallel to studies of phytopathological interest, *I. rickii* has also been the subject of studies to prospect for secondary metabolites and lignocellulolytic enzymes involved in attacking the host and degrading the constituents of wood (Xavier-Santos et al., 2004; Tura et al., 2009; Chen et al., 2014), aiming, above all, at an option sustainable in the bioremediation of recalcitrant compounds. The species is also associated with cultural events in the Wayuu indigenous community, Colombia, in which women apply a layer of chlamydospores to their face, previously treated with sheep tallow to protect themselves from solar radiation (Villalobos et al., 2017). Moreover, taxonomic and phylogenetic aspects are still performed, specially to understand its anamorphic stage.

Although molecular studies carried on different samples confirmed the relationship between anamorphic and teleomorphic phases of *I. rickii*. A preliminary phylogenetic analysis of samples (anamorphic and teleomorphic) collected from different geographical origins showed came from Florida, United States, were separated from those came from Asia, South America and Europe (De Simone et al., 2011; Cui et al., 2014). An interesting aspect to consider that all species from the Florida were collected in the anamorphic stage and the teleomorphic stage has never been recorded in North America. These results suggest the existence of two possible distinct species and are reinforced by morphophysiological, ecological data and climatic conditions (De Simone et al., 2011; Cui et al., 2014). However, studies that analyze isolates from North America and other regions, as well as the holotype of both stages of life, are necessary to confirm the distinction.

## Conclusion

Our study expands the knowledge of geographical distribution of *I. rickii*, including the Cerrado biome, as well as of its structural, physiological characteristics, and its hosts. Although common in tropical and subtropical regions, the specimens described here constitute the first documented records of the species for the Brazilian Cerrado and for the Midwest and Southeast regions, in addition to the first report on *S. molle* within Brazil. The checklist of hosts provided may assist in the development of practices for the control of the pathogen in urban areas with a high incidence of infections. Therefore, it should be mentioned here that, although the present study adds new data to information concerning *I. rickii*, this updated checklist must be considered as a provisional one always waiting for continuous supplementation.

## Data Availability Statement

The original contributions presented in the study are included in the article/supplementary material, further inquiries can be directed to the corresponding author/s.

## Author Contributions

LL-S and SX-S designed this study, collected and analyzed the samples, and wrote the manuscript text. LL-S prepared the figures and tables. AA-A reviewed and edited the manuscript. All authors have read and approved to the published version of the manuscript.

## Conflict of Interest

The authors declare that the research was conducted in the absence of any commercial or financial relationships that could be construed as a potential conflict of interest.

## Footnotes

1 scholar.google.com.br

2 www.scielo.org

3 www.scopus.com

4 www.webofknowledge.com
